# A new species of Andean toad (Bufonidae, *Osornophryne*) discovered using molecular and morphological data, with a taxonomic key for the genus

**DOI:** 10.3897/zookeys.108.1129

**Published:** 2011-06-17

**Authors:** Diego J. Páez-Moscoso, Juan M. Guayasamin, Mario Yánez-Muñoz

**Affiliations:** 1 Museo de Zoología, Escuela de Ciencias Biológicas, Pontificia Universidad Católica del Ecuador, Av. 12 de Octubre y Roca, Aptdo. 17012184, Quito, Ecuador; 2 Centro de Investigación de la Biodiversidad y el Cambio Climático, Universidad Tecnológica Indoamérica, Av. Machala y Sabanilla, Quito, Ecuador; 3 División de Herpetología, Museo Ecuatoriano de Ciencias Naturales, Rumipamba 341 y Av. de los Shyris. Quito, Ecuador; 4 Programa de Maestría en Biología de la Conservación, Pontificia Universidad Católica del Ecuador. Av. 12 de Octubre y Roca, Aptdo. 17012184, Quito, Ecuador

**Keywords:** Andes, Bufonidae, Ecuador, new species, *Osornophryne*, phylogeny

## Abstract

Combining a molecular phylogeny and morphological data, we discovered a new species of *Osornophryne* from the Amazonian slope of the Ecuadorian Andes. Morphologically, the new taxon is distinguished from all others species in *Osornophryne* by having the Toes IV and V longer than Toes I–III, a short and rounded snout with a small rostral papilla, and conical pustules on flanks. The new species previously was confused with *Osornophryne guacamayo*. A taxonomic key is provided for all known species of *Osornophryne*.

## Abstract (es)

Al combinar una filogenia molecular con información morfológica, se descubrió una nueva especie de *Osornophryne* proveniente de la vertiente amazónica de los Andes del Ecuador. Morfológicamente, el nuevo taxón se distingue de los otras especies del género por tener los Dedos IV y V del pie más largos que los Dedos I-III, un rostro corto y redondeado con una pequeña papilla, y pústulas cónicas en los flancos del cuerpo. La nueva especie estaba previamente confundida con *Osornophryne guacamayo*. Se provee de una clave taxonómica para todas las especies de *Osornophryne* reconocidas hasta la fecha.

## Introduction

*Osornophryne* is endemic to the Andes of Colombia and Ecuador, where it occurs in mountain forests and paramo at elevations between 2100 and 4000 m (Ruiz-Carranza and Hernández-Camacho 1976; Gluesenkamp and Guayasamin 2008; Mueses-Cisneros et al. 2010). Currently, *Osornophryne* contains 10 recognized species: *Osornophryne angel* (Yánez-Muñoz et al. 2010), *Osornophryne antisana* (Hoogmoed 1987), *Osornophryne bufoniformis* (Peracca 1904), *Osornophryne guacamayo* (Hoogmoed 1987), *Osornophryne occidentalis* (Cisneros-Heredia and Gluesenkamp 2010), *Osornophryne percrassa* (Ruiz-Carranza and Hernández-Camacho 1976), *Osornophryne puruanta* (Gluesenkamp and Guayasamin 2008), *Osornophryne sumacoensis* (Gluesenkamp 1995), *Osornophryne talipes* (Cannatella 1986) and *Osornophryne cofanorum* (Mueses-Cisneros et al. 2010). The evolutionary relationships between *Osornophryne* and other bufonid genera remain controversial, with some authors arguing a close affinity with *Atelopus* (Coloma 1997; Gluesenkamp 2001), and others supporting a topology in which *Osornophryne* is sister to most other bufonids (Bocxlaer et al. 2010). However, the monophyly of *Osornophryne* is well established by morphological and molecular characters (Ruiz-Carranza and Hernández-Camacho 1976; Coloma 1997; Gluesenkamp 2001; Bocxlaer et al. 2010); among the most conspicuous putative morphological synapomorphies are: six presacral vertebrae, digits almost completely embedded by an extensive membrane, reduced number or lenght of phalanges in hands and feet, absence of stapes and tympanum, urostlyle laterally expanded and broadly fused with sacrum, inguinal amplexus, and direct development (Ruiz-Carranza and Hernández-Camacho 1976; this work; compare with traits in South American bufonids; Pramuk 2006).

Given the complex topography of the Andes and the opportunity for allopatric speciation in areas with similar climatic conditions, it is possible that morphologically similar populations are evolving independently. Herein, we report and describe a new species of *Osornophryne*, previously confused with *Osornophryne guacamayo*.

## Material and methods

### Morphology.

We examined alcohol-preserved specimens from the herpetological collections at Museo de Zoología of the Pontificia Universidad Católica del Ecuador (QCAZ), Escuela Politécnica Nacional (EPN), and Museo Ecuatoriano de Ciencias Naturales (DH-MECN), all based in Quito, Ecuador. Specimens examined are listed in Appendix I. Fingers are numbered preaxially to postaxially from I–IV to facilitate comparison with previous literature dealing with anurans; however, we stress that in an evolutionary perspective anuran fingers should be numbered from II–V, consistent with the hypothesis that Digit I was lost in anurans (Shubin and Alberch 1986; Fabrezi and Alberch 1996). Morphological measurements were taken with digital calipers to the nearest 0.1 mm and are, as follow: (1) snout–vent length (SVL = distance from tip of snout [excluding the proboscis] to posterior margin of vent); (2) tibia length (TIB = length of flexed hind leg from knee to heel); (3) foot length (FL = distance from base of inner metatarsal tubercle to tip of Toe IV); (4) head length (HL = distance from tip of snout to articulation of jaw); (5) head width (HW = greatest width of head measured between jaw articulations); (6) interorbital distance (IOD = shortest distance between medial margins of upper eyelids); (7) upper eyelid width (EW = greatest width of eyelid measured perpendicular to medial axis of skull); (8) internarinal distance (IND = distance between internal borders of nostrils); (9) eye–nostril distance (EN = distance from anterior corner of eye to posterior border of nostril); (10) snout–eye distance (SE = distance from anterior corner of the eye to the tip of the rostrum); (11) eye diameter (ED = distance between anterior and posterior corners of eye); (12) Finger-III length (FIIIL = distance from proximal border of Finger I to distal end of Finger III); (13) Finger-IV length (distance from proximal border of Finger I to distal end of Finger IV); (14) Toe-IV length (TIVL = distance from proximal edge of Toe I to distal tip of Toe IV); (15) Toe-V length (TVL = distance from proximal border edge of Toe I to distal tip of Toe V). Sexual maturity was determinate by the presence of nuptial pads in adult males and convoluted oviducts in adult females. Techniques for clearing and double-staining specimens with Alcian Blue and Alizarin Red were those of Taylor and Van Dyke (1985). Illustrations were made with the aid of a Wild M3B Heerbrugg stereo dissecting microscope equipped with a camera lucida. Osteological terminology is that of Duellman and Trueb (1994), (Fabrezi (1992, 1993), and Trueb (1973, 1993); bufonid osteological character states are illustrated in Pramuk (2006).

### Molecular data.

Fresh liver samples were preserved in 90% alcohol, and stored at –80°C. We used salt-precipitation protocols to extract genomic DNA from ethanol-preserved tissues (M. Fujita, unpubl. data). To amplify the mitochondrial gene 12S, we used the primers MVZ59 and tRNA-val, developed by Graybeal (1997) and Goebel et al. (1999), respectively; Polymerase Chain Reaction (PCR) amplification protocol was, as follows: 1 cycle of denaturation 2 min at 94°C, annealing for 30 sec at 42°C, extension for 1 min at 72°C, 5 cycles of denaturation 30 sec at 94°C, annealing for 30 sec at 42°C, extension for 1 min at 72°C, 22 cycles of denaturation 30 sec at 94°C, annealing for 30 sec at 50°C, extension for 1 min at 72°C, final extension at 72°C was conducted for 5 min. PCR products were visualized in 0.7% agarose gel, and unincorporated primers and dNTPs were removed from PCR products using ExoSap-it purification. Cycle sequencing reactions were conducted by the commercial company Macrogen Inc. Data from heavy and light stands were compared to generate a consensus sequence for each DNA fragment with Sequencer Ver. 4.8. We obtained sequences of 71 specimens, including all the species in *Osornophryne*, except *Osornophryne talipes*, and three outgroup taxa. In addition, sequences were downloaded from GenBank (NCBI). Sequences were initially aligned in Clustal X (Larkin et al. 2007) and adjusted in Mesquite 2.71 (Maddison and Maddison 2009). Best-fit model of molecular evolution was selected in jModeltest 1.1 (Posada 2008) under the Akaike Information Criterion (AIC). Model parameters estimated from jModelTest were used in Bayesian analyses.

### Phylogenetics.

Analyses were conducted using Maximum Parsimony (MP), Maximum Likelihood (ML), and Bayesian Analyses (BA). Parsimony analyses were performed in PAUP (Swofford 2009) using heuristic searches (10,000 stepwise random additions with TBR branch-swapping) and clade support was estimated via 1000 bootstraps with 10 random additions. Maximum likelihood was run in GARLI 0.951 (Zwickl 2006), which uses a stochastic genetic algorithm-like approach to find the topology, branch lengths, and substitution model parameters that maximize the log-likelihood simultaneously (Zwickl 2006). We performed a total of 50 runs to reduce the probability of inferring a suboptimal likelihood solution. Node support was assessed via 1000 bootstrap replicates. For Bayesian analyses, we implemented the model of nucleotide substitution selected as the best fit for the particular dataset according to the Akaike Information Criterion (AIC) in jModeltest 1.1 (Posada 2008). Bayesian analysis of the mitochondrial dataset was performed in Mr Bayes 3.1 (Ronquist and Huelsenbeck 2003). The analysis consisted of 10 million generations and two Markov chains with default heating values. The prior used for the rate matrix was a uniform Dirichlet and no prior information on topology was incorporated. Trees were sampled every 1000 generations; stationarity was assessed by examining the standard deviation of split frequencies and by plotting the –lnL per generation using Tracer v1.4 (Rambaut and Drummond 2007), and trees generated before stationary were discarded as ‘‘burn-in.” Bootstrap values *p* > 70% are considered to indicate strong support (Hillis and Bull 1993, with their caveats). Clades with posterior probabilities *p >* 0.95 are considered strongly supported, but we caution that relatively high posterior probabilities for short internodes (particularly those with low bootstrap values) may be over-estimates of confidence (Alfaro et al. 2003; Erixon et al. 2003).

## Results

**Figure 1.**
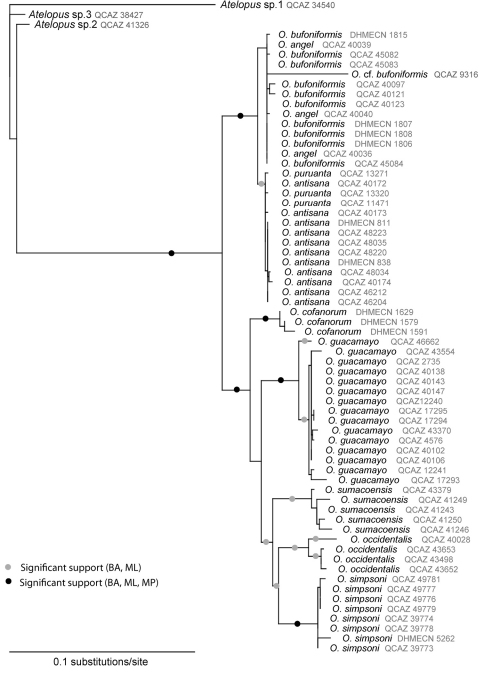
Maximum likelihood phylogeny of the species in *Osornophryne* inferred from the mitochondrial gene 12S (lnL = –1384,3649).

For most of the species and population of *Osonophryne* and three species of *Atelopus*, we obtained a total of 800 bp from the mitochondrial marker 12S rRNA (Table 1). Parameter value estimates for best-fit models for 12S gene generated by jModeltest 1.1 are TIM2 + I (0.001) + G (0.4700). The only taxon for which we could not obtain molecular information was *Osornophryne talipes*, a species that, in Ecuador, is only know from a specimen collected on 02 August 1970 (Cannatella 1986). The different analyses (MP, ML, and AB) are congruent (Fig. 1). The topology resolves most of the relationships among species in *Osornophryne*, and reveals the presence of the previously unrecognized taxon described below.

### Osornophryne simpsoni sp. n.

urn:lsid:zoobank.org:act:9BFBC919-F698-4FDE-8994-9154FF9E99ED

http://species-id.net/wiki/Osornophryne_simpsoni

#### Holotype.

**Figure 2.**
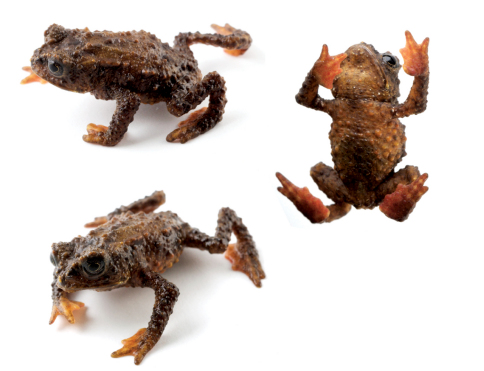
*Osornophryne simpsoni* sp. n. in life (male holotype, QCAZ 49774).

**Figure 3.**
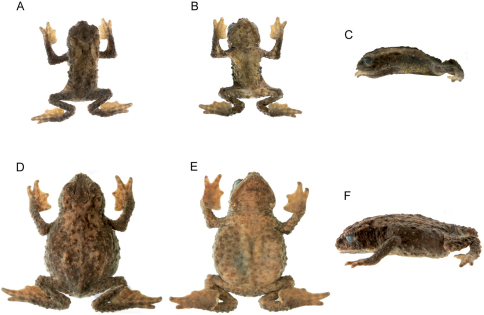
*Osornophryne simpsoni* sp. n. in alcohol. **A–C** Dorsal, ventral and lateral views of holotype, adult male, QCAZ 49774, SVL 20.1 mm **D–F** Dorsal, ventral and lateral views of adult female, DH-MECN 5260, SVL 33.0 mm.

QCAZ 49774 (Figs 2, 3), an adult male near San Rafael-Chontayacu (1°16'34.61"S, 78°4'21.14"W, 2266 m.a.s.l.), Reserve Ankaku-Zona, Río Challuwayacu, Provincia de Pastaza, Ecuador, by Elicio Tapia on 21 October 2009.

#### Paratopotypes:

QCAZ 48781, 49777, 45899 obtained with holotype.

#### Paratypes.

DH-MECN 5660 adult female, DH-MECN 5261, 5263, 5258–59, adult male obtained near Reserva Biológica Río Zuñac (1°20'57.87"S, 78°09'31.37"W, 2250 m.a.s.l.), Parroquia Río Negro, Cantón Baños, Provincia de Tungurahua, Ecuador, by MYM, M. Urgiles y A. Laguna on 17 May 2008; QCAZ 39769 also obtained near Reserva Biológica Río Zuñac, by DJP, A. Narváez, and J. P. Reyes-Puig on 21 January 2009.

**Table 1.** Summary of specimens sequenced of *Osornophryne* and *Atelopus* for the gen 12S and GenBank accession numbers.

Species and museum no.	Locality	Latitude and Longitude	GenBank No.
*Atelopus* sp.			
QCAZ 34540	Limón		JF907488
QCAZ 41326	Zuruni		JF907486
QCAZ 38427	Las Tres Cruces		JF907487
*Osornophryne angel*			
QCAZ 40039	Páramo del Ángel	0°41'15"N, 77°52'46"W	JF907459
QCAZ 40036	Páramo del Ángel	0°41'15"N, 77°52'46"W	JF907458
QCAZ 40040	Páramo del Ángel	0°41'15"N, 77°52'46"W	JF907493
*Osornophryne antisana*			
QCAZ 40172	Páramo de Oyacachi	0°10'34"S, 78°0.6'50"W	JF907453
QCAZ 40173	Páramo de Oyacachi	0°10'34"S, 78°0.6'50"W	JF907485
QCAZ 40174	Páramo de Oyacachi	0°10'34"S, 78°0.6'50"W	JF907484
DH-MECN 838	Salvefaccha	0°13'54"S, 78°0.1'1"W	JF907490
DH-MECN 811	Salvefaccha	0°13'54"S, 78°0.1'1"W	JF907489
QCAZ 46204	Llanganates	1°15'57"S, 78°26'45"W	JF907450
QCAZ 46212	Llanganates	1°15'57"S, 78°26'45"W	JF907449
QCAZ 48035	Llanganates	1°15'57"S, 78°26'45"W	JF907448
QCAZ 48223	Llanganates	1°15'57"S, 78°26'45"W	JF907445
QCAZ 48220	Llanganates	1°15'57"S, 78°26'45"W	JF907446
QCAZ 48034	Llanganates	1°15'57"S, 78°26'45"W	JF907447
*Osornophryne bufoniformis*			
QCAZ 40123	Santa Bárbara	0°38'29"N, 77°31'18.5"W	JF907431
QCAZ 40121	Santa Bárbara	0°38'29"N, 77°31'18.5"W	JF907432
QCAZ 40003	Santa Bárbara	0°38'29.5"N, 77°31'18.5"W	JF907430
QCAZ 45082	Huaca	0°40'15"N, 77°46'11"W	JF907460
QCAZ 45083	Huaca	0°40'15"N, 77°46'11"W	JF907461
QCAZ 45084	Huaca	0°40'15"N, 77°46'11"W	JF907462
DH-MECN 1815	Playón de San Francisco	1°37'43"N, 77°54'35"W	JF907455
DH-MECN 1806	Playón de San Francisco	1°37'43"N, 77°54'35"W	JF907457
DH-MECN 1807	Playón de San Francisco	1°37'43"N, 77°54'35"W	JF907456
DH-MECN 1808	Playón de San Francisco	1°37'42.6"N, 77°54'35"W	JF907454
*Osornophryne cf. bufoniformis*			
QCAZ 9316	Vía Tulcán-Maldonado	0°47'31"N, 77°54'25"W	AF375498
*Osornophryne cofanorum*			
DH-MECN 1591	La Bonita	00°29'19"N, 77°35'11"W	JF907440
DH-MECN 1579	La Bonita	00°29'19"N, 77°35'11"W	JF907439
DH-MECN 1629	La Bonita	00°29'19"N, 77°35'11"W	JF907441
*Osornophryne guacamayo*			
QCAZ 40138	Poblado de Oyacachi	0°10'34"S,, 78°0'50"W	JF907463
QCAZ 40143	Poblado de Oyacachi	0°10'34"S,, 78°0'50"W	JF907464
QCAZ 40147	Poblado de Oyacachi	0°10'34"S,, 78°0'50"W	JF907465
QCAZ 43370	Volcán Sumaco	0°34'11"S, 77°35'39"W	JF907474
QCAZ 4576	Volcán Sumaco	0°34'11"S, 77°35'39"W	JF907491
QCAZ 40106	Cordillera de los Guacamayos	0°37'26.5"S, 77°50'27"W	JF907468
QCAZ 40102	Cordillera de los Guacamayos	0°37'26.5"S, 77°50'27"W	JF907492
QCAZ 43554	Cordillera de los Guacamayos	0°37'26.5"S, 77°50'27"W	JF907467
QCAZ 17295	Volcán Reventador	0°6'43"S, 77°40'44"W	JF907471
QCAZ 17294	Volcán Reventador	0°6'43"S, 77°40'44"W	JF907473
QCAZ 17293	Volcán Reventador	0°6'43"S, 77°40'44"W	JF907472
QCAZ 12240	Río Angel	0°37'26.5"S, 77°50'27"W	JF907469
QCAZ 12241	Río Angel	0°37'26.5"S, 77°50'27"W	JF907470
QCAZ 2735	Jondachi (Río Angel)	0°37'26.5"S, 77°50'27"W	JF907466
QCAZ 46662	Santa Bárbara	0°33'51"N, 77°31'38"W	JF907475
*Osornophryne occidentalis*			
QCAZ 40028	Chilma	0°51'50"N, 78°4'1"W	JF907436
QCAZ 43652	Cuellaje	0°27'30"N, 78°32'43"W	JF907444
QCAZ 43498	Cuellaje	0°27'30"N, 78°32'43"W	JF907443
QCAZ 43653	Cuellaje	0°27'30"N, 78°32'43"W	JF907442
*Osornophryne puruanta*			
QCAZ 13271	Laguna de San Marcos	0°7'36"N, 78°15'22"W	JF907451
QCAZ 13320	Laguna de San Marcos	0°7'36"N, 78°15'22"W	JF907452
QCAZ 11471	Laguna de Puruanta	00°12’ N, 77°57’ W	AF375499.1
*Osornophryne simpsoni*			
QCAZ 49779	Llanganates	1°16'35"S, 78°4'21"W	JF907482
QCAZ 49777	Llanganates	1°16'35"S, 78°4'21"W	JF907477
QCAZ 49781	Llanganates	1°16'35"S, 78°4'21"W	JF907483
QCAZ 49776	Llanganates	1°16'35"S, 78°4'21"W	JF907476
QCAZ 39774	Río Zuñac	1°20'58"S, 78°09'31"W	JF907478
DH-MECN 5262	Río Zuñac	1°20'58"S, 78°09'31"W	JF907480
QCAZ 39778	Rio Zuñac	1°20'58"S, 78°09'31"W	JF907479
QCAZ 39773	Rio Zuñac	1°20'58"S, 78°09'31"W	JF907481
*Osornophryne sumacoensis*			
QCAZ 41243	Volcán Sumaco	0°34'11"S, 77°35'39"W	JF907434
QCAZ 41250	Volcán Sumaco	0°34'11"S, 77°35'39"W	JF907433
QCAZ 41246	Volcán Sumaco	0°34'11"S, 77°35'39"W	JF907437
QCAZ 41249	Volcán Sumaco	0°34'11"S, 77°35'39"W	JF907438
QCAZ 43379	Volcán Sumaco	0°34'11"S, 77°35'39"W	JF907435

#### Diagnosis.

**Figure 4.**
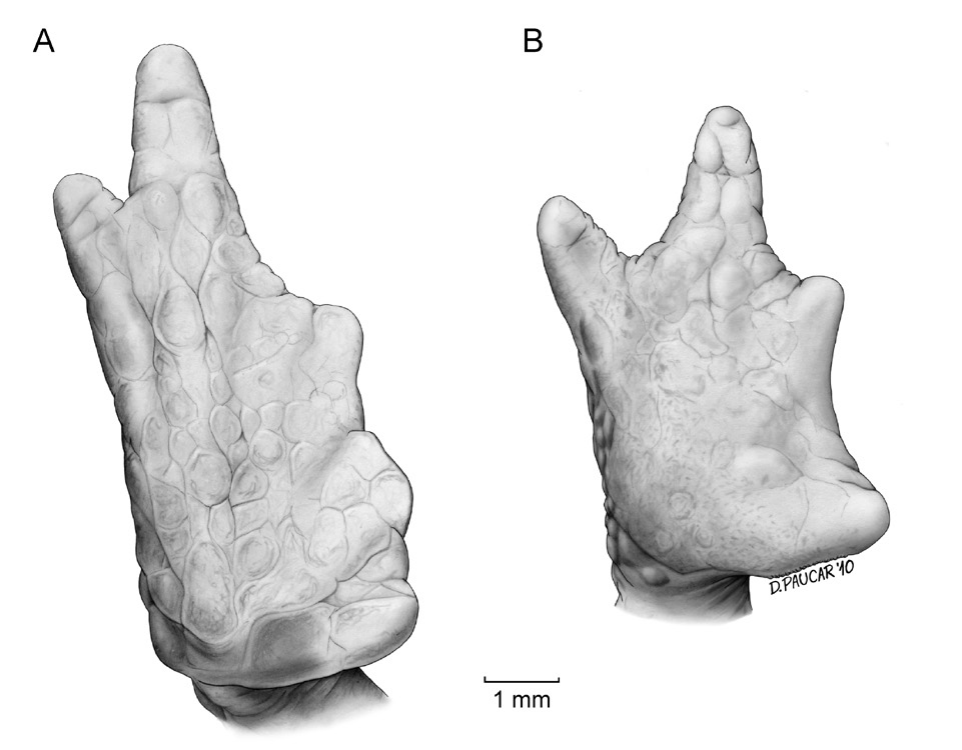
Foot (**A**) and hand (**B**) of *Osornophryne simpsoni* sp. n. (holotype, adult male, QCAZ 49774).

*Osornophryne simpsoni* differs from all other species in *Osornophryne* (except for *Osornophryne guacamayo* and *Osornophryne cofanorum*) by having Toes IV and V longer than Toes I–III (Fig. 4). Morphologically, *Osornophryne simpsoni* is most similar to *Osornophryne guacamayo;* both species have Toes IV and V longer than Toes I–III, pustular dorsal skin, and dark brown dorsal coloration. However, *Osornophryne simpsoni* lacks the conspicuos proboscis present in *Osornophryne guacamayo;* males of *Osornophryne simpsoni* can be distinguished from males of *Osornophryne guacamayo* by having ventral skin with conical pustules (non-conic pustules in *Osornophryne guacamayo*), and light brown to orange conical pustules on the flanks (dark brown to black non-conical pustules in *Osornophryne guacamayo*); the venter of female *Osornophryne guacamayo* is mostly whitish to yellowish with brown marks, whereas that of female *Osornophryne simpsoni* is orange-brown. *Osornophryne cofanorum* differs from *Osornophryne simpsoni* by having its vertebrae and urostlyle coosified with the overlying skin (not co-osified in *Osornophryne simpsoni*) and vertebral neural spines that are visible dorsally (not visible in *Osornophryne simpsoni*); also, males of *Osornophryne cofanorum* have yellow pustules on the tip of the snout, upper eyelid, limbs, and dorsolateral pustular clusters (absent in *Osornophryne simpsoni*). Finally, *Osornophryne simpsoni* is distinguished from its sister species, *Osornophryne occidentalis*, by having a rounded snout in lateral view (protruding in *Osornophryne occidentalis*), brown dorsum with some lighter patches (dark brown dorsum with dark ochre-brown warts in *Osornophryne occidentalis*), orange-brown venter (white in *Osornophryne occidetalis*), and by inhabiting in the Amazonian slopes of the Andes (*Osornophryne occidentalis* is found on the Pacific slopes of the Andes).

#### Species Description.

Ten adult males and one adult female. Females of medium size (SVL = 33.0 mm, *n* = 1); males small (SVL = 17.6–26.1 mm; mean = 21.1 ±2.40, *n =* 10; Table 2). Head length 77.2–95.1% head width; male head width 34.9–40.8% SVL; female head width 37.3% SVL; width of head greater at level of posterior margin of mouth; snout short, rounded, with rostral papilla in dorsal and lateral views; nostrils slightly swollen; each nostril oblique, oval, directed laterally; internarial area concave in males and slightly concave in female; interorbital region with skin co-osified with underlying bone, which has few low tubercles; occipital region mostly flat, but with few bony tubercles and cranial crests in males and females; upper eyelids finely tuberculate in females, with conical tubercles in males; interorbital region wider than the upper eyelid (upper eyelid 73.0–87.5% of interorbital distance in males, *n* = 9; 64.4% in female); outer edge of the eyelid delineated by a continuous row of warts, which are more conical in males than in female; canthus rostralis straight; loreal region slightly concave, with small warts in males and female; pale brown lips; eyes with oval horizontally pupil; infraorbital and postorbital regions with some prominent tubercles of variable size in males and females. Skin of dorsum highly tuberculate, with discontinuous row of conical tubercles starting at level of posterolateral edge of cranium and ending at level of sacrum in males and females; in males, ventral skin with several small pustules and few conical tubercles on gular region and toward the flanks, pustules much denser on chest and abdomen and less conical; in females, ventral skin smooth, with small, non-conical isolated pustules, pustules more numerous on abdomen.

Forelimb long, slender, finely granular, with several larger tubercles extending along inner and outer edges of fingers in males; in females, tubercles smaller than in males. Hand of moderate length, representing 25.0–30.4% (*n* = 10) of SVL in males and 28.5% in female; extensive webbing between fingers (Fig. 4); lengths of fingers in order of increasing length : I < II < IV < III; palms with numerous tubercles; subarticular tubercles not distinguishable; palmar tubercle rounded, thenar tubercle almost undistinguishable.

Hind limbs long and slender; well-defined pustules present on inner and outer edges of fingers in males, females with less pronounced pustules than those in males; tibia and foot, respectively, 32.8–36.9% and 34.9–42.5% of male SVL, and 33.9% and 41.9% of female SVL; webbing between Toes I–III more extensive that webbing between Toes IV–V (Fig. 4); lengths of Toes: I < II < III < V < IV; Toe V much longer than Toe III, soles with numerous tubercles; subarticular tubercles indistinguishable; inner metatarsal tubercle oval. Choanae slightly rounded; adult males lacking vocal sacs; vocal slits absent; nuptial pads on proximal surfaces of Toes I and II, not pigmented; cloacal opening medial to thighs.

#### Coloration in alcohol.

Dorsum, head, forearms, and hind limbs brown to dark brown, with some orange patches; tubercles on upper eyelid, proboscis, and flanks pale yellow. Throat pale yellow; venter cream with brown tubercles.

#### Coloration in life.

Dorsum, head, forearms, and hind limbs dark brown to light brown with some lighter patches; tubercles on upper eyelid, proboscis and flanks orange to yellow. Throat cream yellow, with small dark marks; venter orange-brown.

#### Osteology.

The following osteological description of *Osornophryne simpsoni* is based on a cleared-and-double stained adult male (QCAZ 45899, SVL = 19.5 mm). The osteological description of females was not possible because only one female is known.

Cranium. *Shape and proportions*. The skull is widest posterior to the orbit at the level of the articulation of the maxilla with the quadratojugal. The braincase is broad; at the level of the midorbit, the width of the braincase is about 41.2% of the greatest width of the skull and 26.2% of the medial skull length.

##### Neurocranium.

The neurocranium is formed by five bones—the sphenethmoid, and the paired prootics and exoccipitals. Anteriorly, the neurocranium is completely ossified. A minute septomaxilla is embedded in the anterior nasal capsule cartilage. In dorsal aspect, the cartilaginous planum antorbitale has a perpendicular orientation in relation to the longitudinal axis of the skull. In lateral and ventral views, a broad cartilaginous separation between bony sphenethmoid and prootic is evident. The frontoparietal fontanelle is partially exposed medially between the frontoparietals. Distally, the otic capsules are cartilaginous. Medially, the exoccipitals are slightly separated from one another. The dorsal surface of each prootic is smooth. The epiotic eminences are prominent.

##### Auditory apparatus.

The stapes and tympanic annulus are absent. The operculum is oval and cartilaginous.

##### Dermal investing bones.

**Figure 5.**
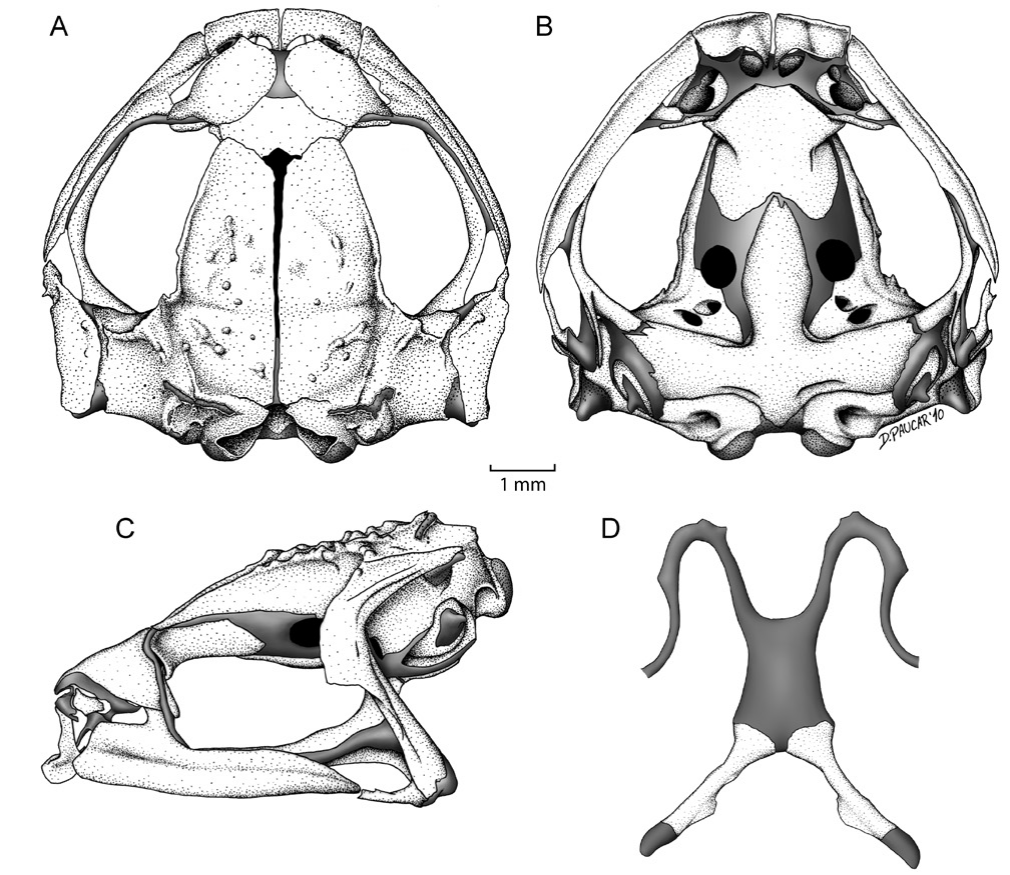
Skull and hyoid of *Osornophryne simpsoni* sp. n., adult male, QCAZ 45899. **A** Dorsal view of skull **B** Ventral view of skull **C** Lateral view of skull **D** Ventral view of hyoid.

Dorsal investing bones are well developed. The nasals are separated from one another and cover most of the nasal capsules dorsally. The maxillary process of the nasal overlaps the pars fascialis of the maxilla to form a bony anterior margin of the orbit. The frontoparietals are well developed and have a narrow separation between one another along its longitudinal axis. The posteriormedial margin of each frontoparietal contacts the exoccipital, but is not fused to it. Posterolateraly, each frontoparietal bears a bony extension that reaches the epiotic eminence. Each frontoparietal has a lamina perpendicularis that is narrow anteriorly and greatly expanded posteriorly (Fig. 5C). The dorsal surface of each frontoparietal bears small, bony tubercles that are visible externally; the tubercles seem to be co-ossified with the overlying skin.

##### Ventral investing and palatal bones.

The parasphenoid has the shape of an inverted T. The broad cultriform process extends anteriorly to about the mid-level of the orbit, where it is narrowly separated from the posterior border of the sphenethmoid. The cultriform process reaches its maximum width at a level that is coincident with the posterior margin of the optic fenestra. The parasphenoid alae are robust, investing the cartilaginous floor of the otic capsule anterior to the exoccipitals; the length of each ala is 61.8% the length of the cultriform process. A broadly acuminate posteromedial process of the parasphenoid terminates just anterior to the margin of the foramen magnum. The vomers are small, arcuate, broadly separated bones that support the medial margins of the choanae; the bones are unornamented, edentate, and lack dentigerous processe; the prechoanal ramus of the vomer is especially short. The neopalatine is short and narrow; medially, it reaches the anterolateral margin of the sphenethmoid; medially, the neopalatine does not contact the maxilla (Fig. 5B).

##### Maxillary arcade.

The premaxillae and maxillae lack teeth. The arcade is complete and has a tenuous articulation with the short quadratojugals. The pars palatinae of the premaxillae are broad. The premaxilla bears two palatine processes, a narrow longer medial and a broad lateral process. There is a simple, juxtaposed articulation between the anterior end of the maxilla and the premaxilla. The pars facialis of the maxilla is well-developed anteriorly, covering the the posterior region of the olfactory capsule; also, the pars facialis has a well-developed preorbital process, which covers most of the planum antorbitale (Fig. 5B, C).

##### Suspensory apparatus.

The tridiate pterygoid bears a slightly curved anterior ramus that is orientated anterolaterally toward the maxilla, with which it articulates. The pterygoid is in close proximity to the maxilla and the narrow space between them is filled by the pterygoid cartilage. The medial and posterior rami of the pterygoid are about equal in length; however, the medial ramus is more robust than the posterior. The lateral end of the medial ramus overlaps the lateral edge of the prootic. The squamosal has the shape of an inverted L; the zygomatic ramus is almost absent, whereas the otic ramus is long and almost reaches the posterior end of the skull. The otic ramus overlaps the lateral margin of the crista parotica slightly. The ventral ramus invests the lateral surface of the palatoquadrate, and articulates with the quadratojugal (Fig. 5A, C); along its anterior margin, the ventral ramus has a conspicuos flange, which extends along the upper border of the otic ramus (Fig. 5C).

##### Hyoid.

The width of the cartilaginous hyoid corpus is narrower than its medial length (width 63.1% of length). The anterolateral and posterolateral processes of the hyoid are absent. The bony posteromedial processes are slightly expanded proximally; each process has a bony flange along the posteromedial margin. The hypoglossal sinus is broadly U-shaped. The hyalia are simple and lack any processes (Fig. 5D).

##### Postcranium. Vertebral column

**Figure 6.**
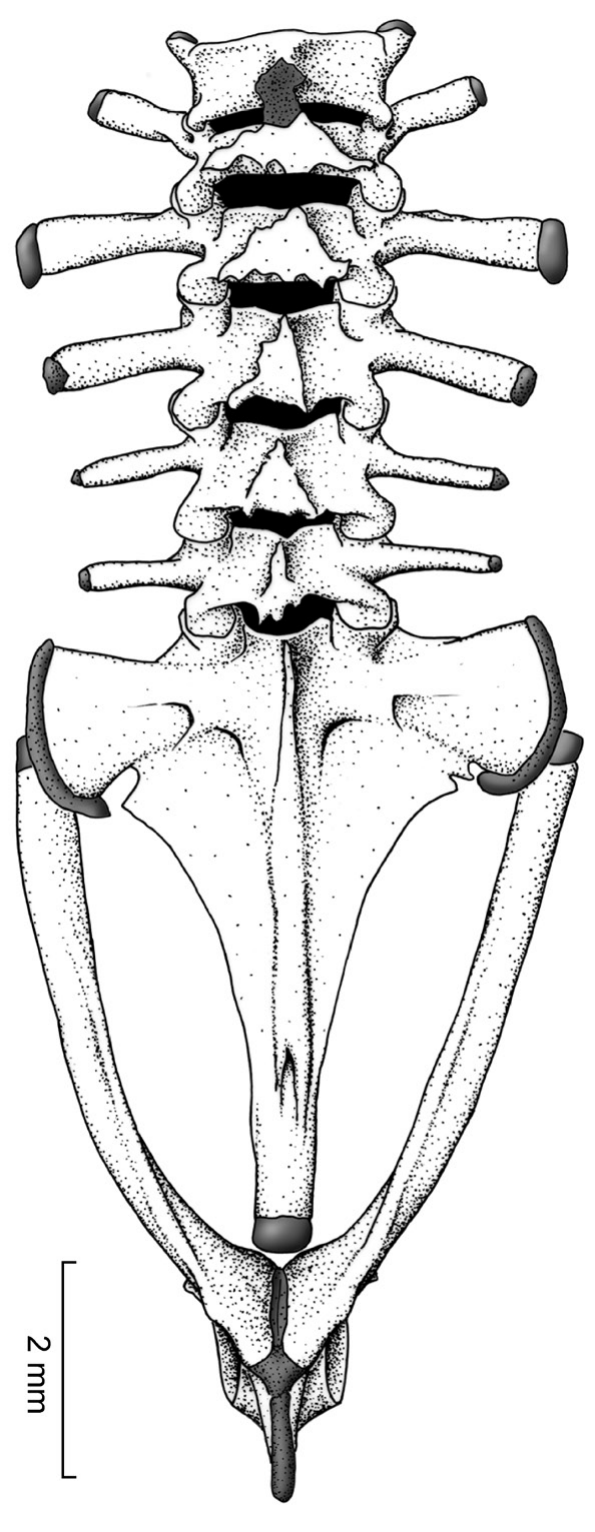
Vertebral column of *Osornophryne simpsoni* sp. n. in dorsal view; adult male, QCAZ 45899.

There are six prepresacral vertebrae. Presacrals I and II are not fused and are notably shorter than Presacrals III–VI. The vertebral profile in decreasing order of overall width of bony parts is: Sacrum > III > IV > V > VI > II > I. Presacral I, or the atlas, lacks transverse processes. All presacrals are non-imbricate. The transverse processes of Presacral II have a anterolateral orientation, Presacrals III–V have a slightly posterolateral orientation, and Presacral VI is approximately perpendicular to the longitudinal axis of the body. The bony sacral diapophyses are broadly expanded; posteriorly, the sacrum is broadly fused with the urostyle, which is greatly expanded laterally. The urostyle bears a well-developed dorsal crest throughout most of its length (Fig. 6).

##### Pectoral girdle.

**Figure 7.**
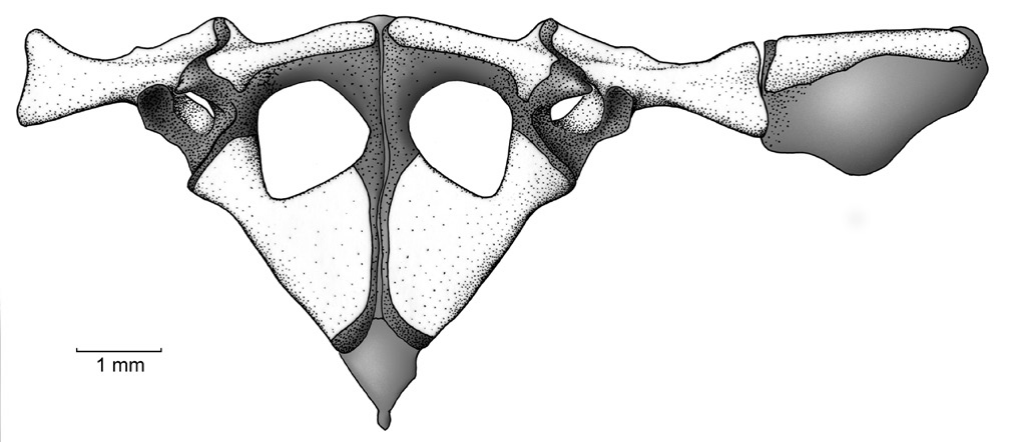
Pectoral girdle of *Osornophryne simpsoni* sp. n. in ventral view; adult male, QCAZ 45899.

The clavicles have a slight orientation, with the medial tips distinctly separated from one another and located at about the same level of the anterolateral end of the clavicle, which articulates with the pars acromialis of the scapula (Fig. 7). The coracoid is notably stout, with the sternal end having a moderate expansion and the sternal end being heavily expanded (sternal end 45% of glenoid end); the inner edge of coracoid has an angle of about 45˚, wheras the external edge is straight (no angle). The pectoral fenestra is has a tringular shape, in which the base is anteriorly convex. The scapula is moderately long with a prominent pars acromialis that is separated from the pars glenoidalis; the leading and posterior edges of the scapula are slightly concave. The suprascapula is mostly cartilaginous, but it is mineralized at both ends, with the ossified cleithrum apparent as a slender bone along the leading edge of the suprascapular blade and with a proximal end that is wider than its distal end. The sternum is small and completely cartilaginous; it contacts the epicoracoid cartilage, which is extensive, and the posterior margin of the coracoid. The omosternum is absent.

##### Pelvic girdle.

The long, slightly concave, and slender ilial shafts bear small dorsal crests, which extend from the anterior third to the posterior end of the shafts (Fig. 6). The ilial prominence is broad and low; the pubes is highly mineralized.

##### Manus and pes.

**Figure 8.**
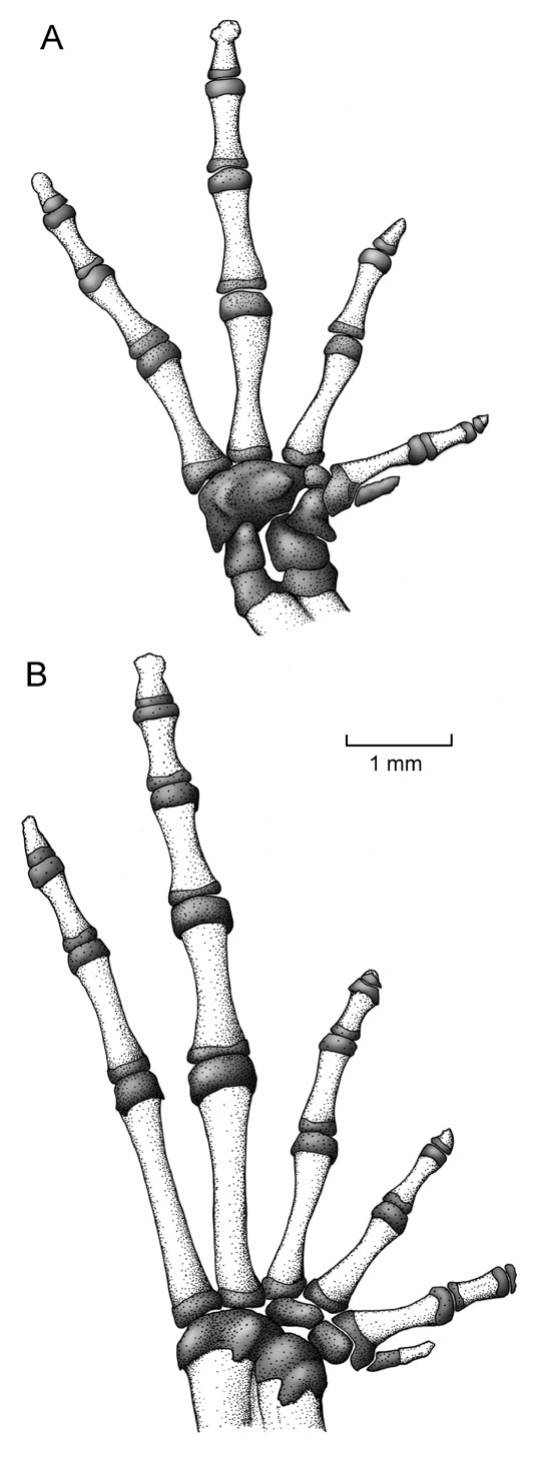
Osteology of hand and foot of *Osornophryne simpsoni* sp. n. in ventral view; adult male, QCAZ 45899.

The phalangeal formulae for the hand and foot are standard—i.e., 2-2-3-3 and 2-2-3-4-3, respectively; however, the distal phalange of Finger I, Toe I, and Toe III are greatly reduced and formed mostly by cartilage (Fig. 8). Relative length of fingers, in increasing order, is: I-II-IV-III, and of the foot is: I-II-III-V-IV. The carpus is composed of a radiale, ulnare, Element Y, Carpal 2, and a large postaxial assumed to represent a fusion of Carpals 3–5. Element Y is about 3 times the size of Carpal 2, and the prepollex is an elongated cartilage. The terminal phalanges are acuminate, except Finger III that is slightly T-shaped. The tarsus is composed of two tarsal elements, presumably Tarsal 1 and Tarsal 2 + 3. The prehallux is presented by a proximal mineralized cartilage element associated with a small bony element.

#### Etymology.

The specific name *simpsoni* is a patronym for Dr. Nigel Simpson in recognition for his continual efforts in protecting the Andean cloud forests of Ecuador. Dr. Simpson is a collaborator of two of the most important conservation NGOs in Ecuador, EcoMinga Foundation (www.ecominga.net) and Jocotoco Foundation (www.fjocotoco.org). As the common name of the species, we suggest “Simpson’s Plumb Toad.” In Spanish, we suggest the name “Osornosapo de Simpson.”

#### Distribution and conservation.

**Figure 9.**
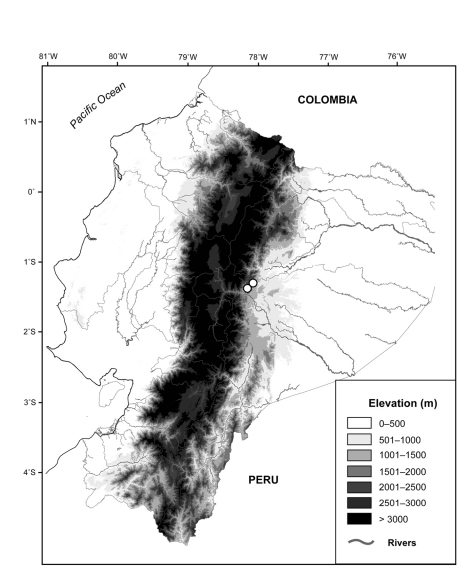
Distribution of *Osornophryne simpsoni* sp. n. (white circles) in Ecuador.

*Osornoprhyne simpsoni* is only known from the type locality and surrounding areas, Reserva Zuñac (1°20'58"S, 78°09'31"W) and Reserve Ankaku-Zona (1°16'35"S, 78°4'21"W; Fig. 9). These localities are included in the Bosque de Niebla Montano (Montane Cloud Forest) according to the classification proposed by Valencia et al.(1999). Vegetation is dominated by Clusia spp. trees. All individuals of *Osornophryne simpsoni* have been found on leaves of bromeliads and ferns during the night. Simpatric anurans include *Pristimantis altamis*, *Pristimantis bicantus*, *Pristimantis imcomptus* and *Pristimantis galdi*. Following the IUCN (2001) criteria, we consider *Osornophryne simpsoni* as Data Deficient; however, it is likely that *Osornophryne simpsoni* has a restricted distribution, as observed in other *Osornophryne* species.

#### Discussion.

It has become increasingly evident that lineage independence is not always accompanied by morphological change when ecological conditions remain similar (Wiens 2004). Therefore, combining different sources of data in the process of species discovery increases the probabilities of revealing evolutionary species (sensu Simpson 1961; Wiley 1978; Padial et al. 2009). The discovery of *Osornophryne simpsoni* represents a good example of such approach. The phylogeny presented in Figure 1 shows some interesting issues that will need further research. For example, given the current gene sampling, there is no genetic differentiation between *Osornophryne angel* and *Osornophryne bufoniformis;* similarly, *Osornophryne antisana* and *Osornophryne puruanta* are not reciprocally monophyletic, although they have conspicuous morphological differences (e.g., body size). Last, within *Osornophryne guacamayo*, there are two genetically distinctive populations that might represent evolutionary species.

**Table 2.** Morphometrics of adult males and female of *Osonophryne simpsoni* sp. n.

Museum number	Sex	SVL	HL	HW	FL	TIB	IOD	EW	IND	IND	FIIIL	FIVL	LM	LF	TL	EN	ED
DH-MECN 5260	Female	33.0	9.5	12.3	13.8	11.4	5.9	3.8	3.1	3.1	8.4	9.4	8.3	13.0	11.2	2.8	3.2
QCAZ 49774	Male	20.1	7.2	8.2	8.4	6.8	3.4	2.8	2.3	2.3	5.3	5.7	5.4	8.0	7.2	1.7	2.9
QCAZ 45899	Male	19.5	6.4	7.6	7.0	5.7	3.7	2.7	1.9	1.9	4.5	5.2	4.9	6.6	6.5	1.7	2.8
QCAZ 49777	Male	17.6	5.9	6.7	7.0	5.6	3.2	2.8	2.0	2.0	4.2	4.6	4.4	6.3	5.9	1.5	2.8
QCAZ 49781	Male	18.6	6.0	7.0	7.1	5.6	3.1	2.6	2.1	2.1	4.6	5.0	4.5	6.5	6.1	1.6	2.1
QCAZ 39774	Male	23.2	7.7	8.1	8.5	7.4	4.0	1.8	2.1	2.1	5.3	5.8	5.3	7.6	7.9	1.9	2.6
QCAZ 39769	Male	26.1	8.2	9.4	9.1	7.7	4.0	3.0	3.0	3.0	6.3	6.6	5.8	8.9	8.8	2.0	2.7
DH-MECN 5261	Male	21.5	7.2	8.0	8.7	7.4	4.1	3.2	2.2	2.2	5.7	6.2	5.4	7.6	7.1	1.9	4.1
DH-MECN 5259	Male	21.4	6.8	8.1	9.1	7.7	4.0	3.1	2.6	2.6	5.8	6.5	5.8	9.3	7.9	2.0	4.0
DH-MECN 5258	Male	21.2	7.2	8.4	8.1	6.8	3.8	2.8	2.6	2.6	5.3	5.8	5.4	8.8	7.6	2.1	3.8
DH-MECN 5263	Male	21.3	6.8	8.0	8.8	7.5	3.6	3.0	2.7	2.7	5.5	5.8	5.3	7.9	7.5	1.8	3.6

## Key to the species of *Osornophryne*

**Table:** 

1	Toe V longer than Toes I–III (Fig. 4A)	2
–	Toe V shorter than Toes I–III	4
2	Vertebrae and urostyle co-ossified with overlying skin; in life, males with yellow pustules on upper eyelid and tip of snout	*Osornophryne cofanorum*
–	Vertebrae and urostyle not coossified with overlying skin; males lacking yellow pustules on tip of snout	3
3	Head acuminate, with a long proboscis (Figs 10, 11); dorsal skin lacking conical tubercles in most populations (except population from Volcan Sumaco); dorsum dark brown to black, sometimes with grayish-yellow dorsolateral stripes	*Osornophryne guacamayo*
–	Head with short and round snout, with small papillae at tip (Figs 10, 11); dorsal skin with conical tubercles; dorsum lacking dorsolateral stripes (Fig. 2)	*Osornophryne simpsoni*
4	Dorsum covered with numerous round pustules of different sizes (lacking space among pustules)	5
–	Dorsum with sparsely distributed pustules (space among pustules clearly evident)	6
5	Female dorsal skin highly tuberculate, with prominent dorsolateral ridges; flanks with large rounded pustules; males and females with prominent occipital ridges; in males, head acuminated to subacuminated in lateral view (Figs 10, 11)	*Osornophryne angel*
–	Female dorsal skin highly tuberculate, with faintly defined dorsolateral ridges; flanks with scattered and small pustules; males and females with low (or lacking) occipital ridges; in males, head rounded or truncated in lateral view (Figs 10, 11)	*Osornophryne bufoniformis*
6	Dorsolateral, occipital, and pelvic ridges separated by smooth skin	7
–	Dorsolateral, occipital, and pelvic ridges separated by flat pustules	9
7	Males and females large (in males, SVL > 23.5 mm; in females, SVL > 40 mm); males and females with a continuous dorsolateral ridges, and acuminate snout in dorsal view (Figs 10, 11)	8
–	Males and females small (in males, SVL < 19.0 mm; in females, SVL < 30 mm). Males and females with discontinuous dorsalateral ridges	*Osornophryne antisana*
8	Males and females with prominent dorsolateral, occipital, and pelvic ridges	*Osornophryne talipes*
–	Females with comparatively lower dorsolateral, occipital, and pelvic ridges (males unknown)	*Osornophryne puruanta*
9	Females with yellow, orange, or white venter in life	10
–	Females with blue to silver venter in life; males with a yellow to orange papillae at tip of snout (Figs 10, 11)	*Osornophryne sumacoensis*
10	Females with yellow to orange venter in life; males and females lacking dorsolateral ridges	*Osornophryne percrassa*
–	Females with yellow to white venter; males and females with clearly defined dorsolateral ridges	*Osornophryne occidentalis*

**Figure 10.**
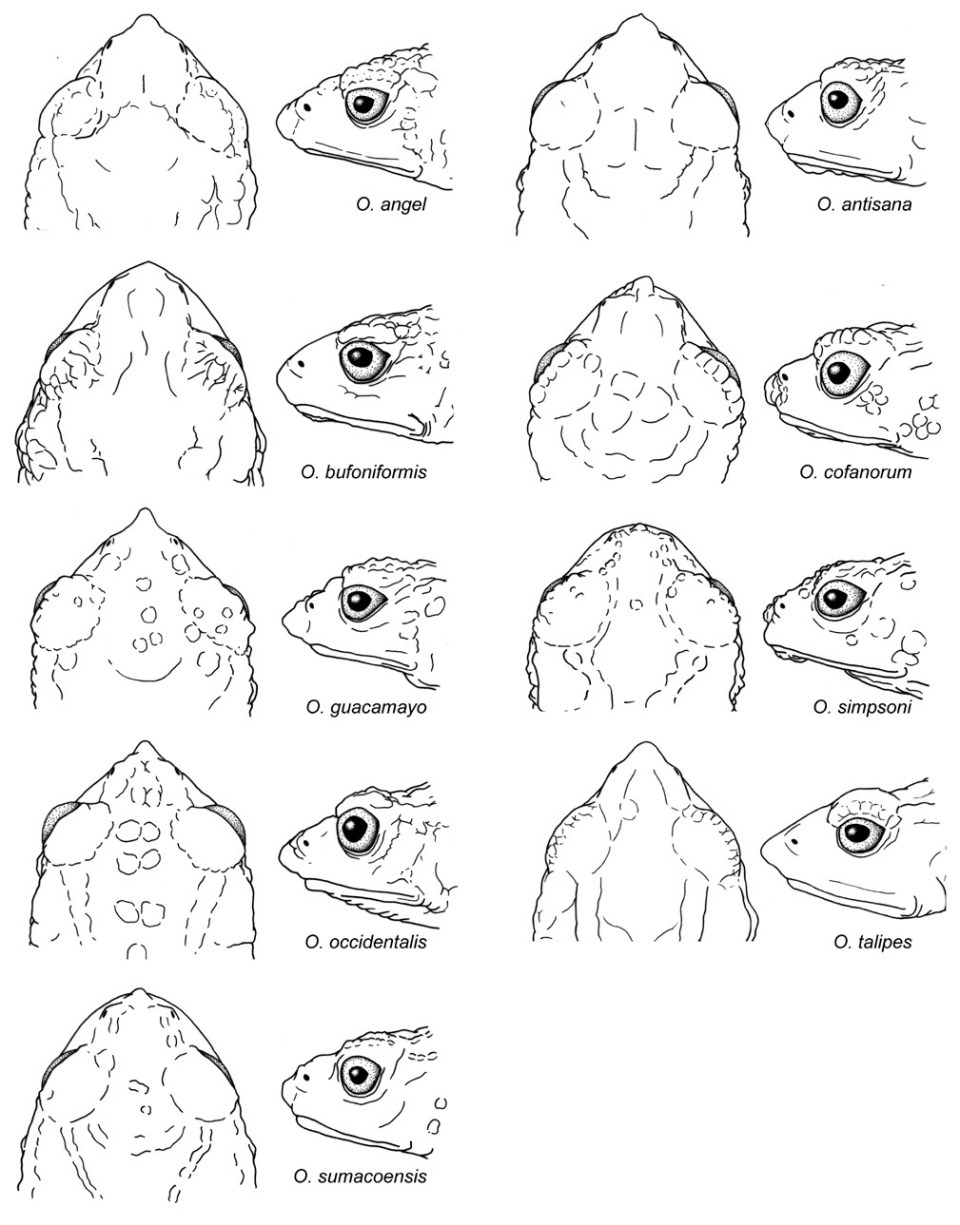
Head shape in dorsal and lateral views of *Osornophryne* males. Illustrated species are: *Osornophryne angel*, QCAZ 40048; *Osornophryne antisana*, QCAZ 48209; *Osornophryne bufoniformis*, QCAZ 45084; *Osornophryne cofanorum*, DH-MECN 6248; *Osornophryne guacamayo*, QCAZ 40106; *Osornophryne simpsoni*, QCAZ 49774; *Osornophryne occidentalis*, QCAZ 43529; *Osornophryne sumacoensis*, QCAZ 41246; *Osornophryne talipes*, ICN 12256. Not drawn at scale.

**Figure 11.**
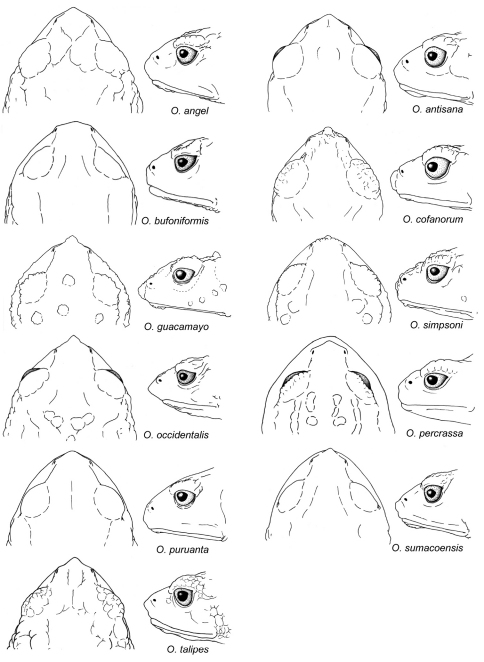
Head shape in dorsal and lateral views of *Osornophryne* females. Illustrated species are: *Osornophryne angel*, QCAZ 43560; *Osornophryne antisana*, QCAZ 48221; *Osornophryne bufoniformis*, QCAZ 40122; *Osornophryne cofanorum*, DH-MECN 6194; *Osornophryne guacamayo*, QCAZ 26047; *Osornophryne simpsoni*, DH-MECN 5260; *Osornophryne occidentalis*, QCAZ 43498; *Osornophryne percrassa*, ICN 319; *Osornophryne puruanta*, QCAZ 11471; *Osornophryne sumacoensis*, QCAZ 41244; *Osornophryne talipes*, EPN 2823. Not drawn at scale.

## Supplementary Material

XML Treatment for Osornophryne simpsoni

## Appendix I: Examined Specimens.

*Osornophryne angel:* Carchi: El Voladero (0°41'15"N, 77°52'46"W), DH-MECN 4617, 4606, 4629–30, 4626, 6079, QCAZ 40030–31, 40040, 40043–49.

*Osornophryne antisana*: Napo: Oyacachi (0°10'34"S, 78°0.6'50"W, 3913 m) QCAZ 40172, 40174; Salvefaccha (0°13'54"S, 78°0.1'01"W, 3900 m) EPN 8937. Tungurahua: Llanganates (1°15'57"S, 78°26'45"W, 3600 m), QCAZ 515, 46282, 48231, 46207, 46204–5, 48230, 48487, 48233–36, 48222, 48215, 48217, 48213, 46212.

*Osornophryne bufoniformis*: Carchi: Huaca (0°40'15"N, 77°46'11"W, 3780 m) QCAZ 45082–4, 45080–81, 43351; El Chamizo QCAZ 14597–98; Tulcán-Maldonado (0°47'31"N, 77°54'25"W, 3817 m) QCAZ 735, 9316–17. Sucumbios: El Playón de San Francisco (00°36'44"N, 77°40'13"W, 3400–4100 m), DH-MECN 6067–70, 6073, 6079, 6082; La Bonita (00°29'19"N, 77°35'11.4"W, 2614 m) QCAZ 46640–43; Santa Bárbara (0°38'29.47"N, 77°31'18.55"W, 2700 m), QCAZ 40003, 40122, 14080–81, 40118–19.

*Osornophryne cofanorum*: Sucumbios: La Bonita (0°29'19"N, 77°35'11"W, 2614 m), DH-MECN 6192, 6194, 6205, 6214, 6219, 6232, 6237, 6243, 6250, 6296–97, 6300, 6303–04, 6315, 6325, 6328, 6334, 6337, 6339.

*Osornophryne guacamayo*: Napo: Cordillera de los Guacamayos (0°37'26.5"S, 77°50'27.09"W, 2238 m), QCAZ 3266, 4889, 9894, 12245, 12249, 13260, 12240, 13259, 26047, 26049, 39081, 33197, 10457, 40106, 40111, 10465, 12241, EPN 6822, 6821, 7438, 7806; Oyacachi (0°15'25"S, 77°57'53"W, 2253 m), QCAZ 40158, 40169, 40138, 40143, 40160, 40148, 40137; Volcán Sumaco (0°34'11"S, 77°35'39"W, 2479 m), QCAZ 41211, 41206, 41210, 41194, 41207, 41229, 41196, 41223, 41203, 41190, 43378, 43370, 43377, 43551–54, 43557, 43550, 43557–59. Sucumbios: Santa Bárbara (0°33'51.4"N, 77°32'50"W, 2388 m), QCAZ 46661.

*Osornophryne occidentalis:* Carchi: Chilma Bajo (0°51'50"N, 78°4'01"W, 2237 m), QCAZ 40028. Imbabura: Rosario (0°27'29"N, 78°32'50"W, 2296 m) QCAZ 36894, 43498, 43649, 43647, 10141, 43652, 43529, 43650, 43646, 43651, 43653. Pichincha: Guarumos (00°02'S, 78°39'W, 2550–2600 m) EPN 1239.

*Osornophryne puruanta*: Imbabura: Laguna de Puruanta (00°12'N, 77°57'W, 3000–3500 m) QCAZ 11471, 7685. Pichincha: Laguna de San Marcos (0°7'36"N, 78°15'22"W, 3834 m), EPN7081–83, QCAZ 13271.

*Osornophryne sumacoensis*: Napo: Volcán Sumaco (0°34'11"S, 77°35'39"W, 2479 m), QCAZ 41247–50, 41243–54, 41233–34, 43379, 4574.
